# Multiform Informed Machine Learning Based on Piecewise and Weibull for Engine Remaining Useful Life Prediction

**DOI:** 10.3390/s23125669

**Published:** 2023-06-17

**Authors:** Shuang Zhou, Yunan Yao, Aihua Liu, Fan Wang, Lu Chen, Ruolan Xiong

**Affiliations:** 1 School of Transportation and Logostics Engineering, Wuhan University of Technology, Wuhan 430063, China; soso_zs@whut.edu.cn; 2 School of Naval Architecture, Ocean and Energy Power Engineering, Wuhan University of Technology, Wuhan 430063, China; ynyao@whut.edu.cn (Y.Y.); 319923@whut.edu.cn (F.W.); 331651@whut.edu.cn (L.C.); 331704@whut.edu.cn (R.X.); 3 Sanya Science and Education Innovation Park, Wuhan University of Technology, Hainan 572024, China

**Keywords:** Weibull function, Piecewise, informed machine learning, remaining useful life prediction, predictive health management

## Abstract

Informed machine learning (IML), which strengthens machine learning (ML) models by incorporating external knowledge, can get around issues like prediction outputs that do not follow natural laws and models, hitting optimization limits. It is therefore of significant importance to investigate how domain knowledge of equipment degradation or failure can be incorporated into machine learning models to achieve more accurate and more interpretable predictions of the remaining useful life (RUL) of equipment. Based on the informed machine learning process, the model proposed in this paper is divided into the following three steps: (1) determine the sources of the two types of knowledge based on the device domain knowledge, (2) express the two forms of knowledge formally in Piecewise and Weibull, respectively, and (3) select different ways of integrating them into the machine learning pipeline based on the results of the formal expression of the two types of knowledge in the previous step. The experimental results show that the model has a simpler and more general structure than existing machine learning models and that it has higher accuracy and more stable performance in most datasets, particularly those with complex operational conditions, which demonstrates the effectiveness of the method in this paper on the C-MAPSS dataset and assists scholars in properly using domain knowledge to deal with the problem of insufficient training data.

## 1. Introduction

Predictive health management (PHM) is gaining popularity in academia and industry as a means of improving system reliability, reducing safety events, and lowering maintenance costs [1]. One of the fundamental technologies of PHM is RUL prediction, which analyzes the health state of equipment using condition monitoring (CM) data and serves as the foundation for equipment health management and maintenance. Collecting multi-source sensor data for CM and predicting the health state of complex equipment (e.g., aero-engines) has become one way to increase equipment dependability and intelligence as a result of the development of the Industrial Internet of Things (IIOT). While reducing the expenses of equipment repair, accurate RUL prediction can ensure safe aircraft flight. The two primary categories of current RUL prediction techniques are model-based methods and data-driven methods [2]. Model-based methods use mathematical descriptions like algebraic and differential equations to model the degradation process of the system. It is quite challenging to create a realistic physical model of aero-engines because of their great degree of structure complexity and the sophisticated interactions between components. The size of aero-engine monitoring data has gradually grown in recent years due to advancements in sensor technology, and many academics have invested in data-driven methods.

Because it can achieve end-to-end prediction from CM data to RUL without understanding the internal structure of the engine and system interactions, machine learning has emerged as a popular research topic among data-driven approaches in recent years. Many shallow ML methods, such as support vector machine (SVM) [3], random forest (RF) [4], deep belief network (DBN) [5], and artificial neural network (ANN) [6] have been applied to RUL prediction and achieved good prediction results. Tian [7] added a training mechanism to the ANN training process to achieve more accurate predictions. Zhang et al. [8] proposed a multi-objective deep belief network (MODBN) for concurrently evolving multiple DBNs to build an RUL integration model. Shen et al. [9] address the issue of insufficient source degradation metrics by referencing intermediate domains in the transfer model SVM.

However, the above ML algorithms are poor at learning and extracting abstract features, making them difficult to adapt to the massive data processing scenario in IIOT. ML evolves from shallow to deep learning aspects, such as convolutional neural networks (CNNs), recurrent neural networks (RNNs), deep neural networks (DNNs), and so on. Li et al. [10] constructed a novel deep CNN model to achieve the RUL prediction of an aero-engine without the a priori knowledge of signal processing. Yang et al. [11] built a double convolutional neural network (DCNN) to identify the initial fault points and perform RUL prediction.

RNN enhances the network structure with self-feedback nerves and excels at handling temporal inputs. The RUL of the device with the same degradation pattern was determined by Yu et al. [12] using a similarity-based health index curve matching technique. Two models, the deep Weibull model (DW-RNN) and the multi-task learning model (MTL-RNN), were created by Aggarwal et al. [13] to study the underlying distribution and potential failure dynamics of an aero-engine, respectively.

RNN has issues such as gradient explosion or disappearance during back-propagation, and it is difficult to handle temporal inputs with long-term dependencies. The long short-term memory (LSTM) network introduces cell states to store long-term memory, which is a good way to address the issue [14]. Bi-directional LSTM networks (Bi-LSTM) can extract more representative salient features from data and produce more accurate and consistent prediction results [15].

When the length of the input sequence exceeds a certain threshold, the LSTM’s memory declines, whereas the self-attention (SA) mechanism can learn time series information of any length and effectively extract spatial and temporal features from the sequence data [16]. To implement weighted sensor sequences and time steps, Song et al. [17] built a distributed attention-based temporal convolutional network (DATCN) prediction model. Zhang et al. [18] proposed a transformer-based dual aspect attention mechanism (DAST) for predicting RUL, in which a parallel attention structure allowed for the simultaneous extraction of features from different sensors and time steps. Narwariya et al. [19] divided the multi-dimensional time series data into meaningful subsets by modeling the internal modular structure of a turbine engine with a gated graph neural network (GGNN).

**Figure 1:**
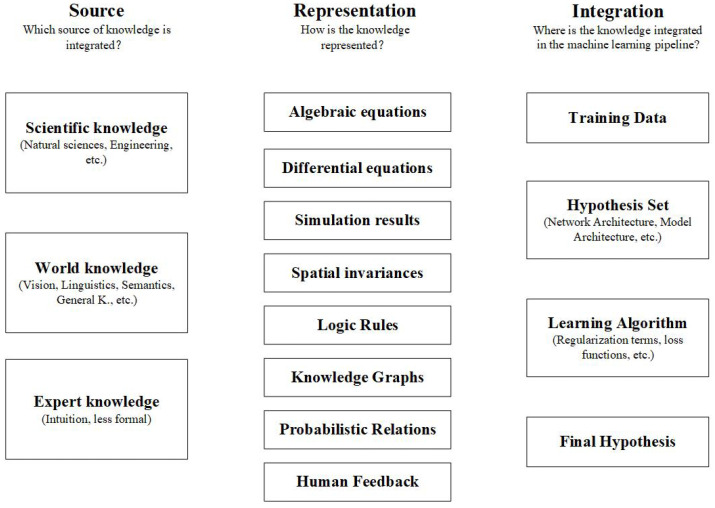
Pathways for IML proposed by Rueden et al. [20].

ML is a useful tool for PHM solutions. However, ML-based approaches can reach their limits or produce unsatisfactory results in cases where the data are insufficient or of poor quality, and there is an increasing requirement for models to be interpretable as ML algorithms get more complicated. Research on how to enhance ML models by incorporating external knowledge into the learning process has been motivated by the aforementioned issues. To enhance ML models, a variety of knowledge domains can be used, such as well-validated equations, models, or techniques. After conducting a systematic and comprehensive review of the literature on how to integrate external knowledge into the ML process, Rueden et al. [20] proposed informed machine learning as an umbrella term for the aforementioned approaches to differentiate it from the traditional ML. In terms of knowledge sources, knowledge representation, and knowledge integration, they developed a detailed classification framework for IML. Figure 1 depicts the various paths of IML. IML can improve predictive performance and help overcome obstacles such as limited or poor-quality data in the domain of PHM [21]. Following Figure 1, the remainder of this paper will introduce IML and its application in the PHM domain in terms of knowledge source, knowledge representation, and knowledge integration.

Knowledge source is the origin of the knowledge integrated into ML, and it is classified into three types: scientific knowledge, world knowledge, and expert knowledge. Scientific knowledge is derived from a wide range of engineering disciplines. Wang et al. [22] proposed a cross-physical data fusion (CPDF) scheme in which a physically based tool-cutting model explored information hidden in unlabeled samples to eliminate physical inconsistencies present in traditional data-driven models, illustrating one approach to scientific knowledge application in ML. Sobie et al. [23] produced data for ML algorithms by using information from a high-resolution simulation of roller bearing dynamics. Baseman et al. [24] used expert knowledge of the spatial interdependencies between storage devices to predict the likelihood of faults in high-performance computing memory. Expert knowledge exists in the form of intuitive and tacit knowledge, which, while less formalized than scientific knowledge, has some room for exploration in PHM.

The core module of IML classification describes how knowledge is formally expressed. Figure 1 illustrates how knowledge can be expressed in several ways. For the purpose of establishing the correlation between fracture length and blade intrinsic frequency, Ellis et al. [25] used ANSYS SMART to model blade crack growth and gather simulation data, such as crack size and expansion path. Berri et al. [26] created a ML model for RUL prediction of airplane brakes using a high-fidelity simulation result. In order to estimate the RUL of high-speed railroad equipment, Zang et al. [27] proposed a model-based and data-driven prediction method that makes use of simulation data and forward neural networks.

Rueden’s paper describes knowledge integration in terms of four aspects: training data, hypothesis set, learning algorithm, and final hypothesis [20]. As shown by Wang et al. [22], Sobie et al. [23], and Ellis et al. [25], a simulation result is knowledge that exists outside the ML domain, so training data can be augmented by simulation results. Integrating knowledge into the hypothesis set is more common; for example, knowledge can be achieved by choosing a model structure where the network architecture is designed considering knowledge elements. Lu et al. [28] embedded linear structures as linear activations in a neural network and eventually used the network to evaluate product quality. The final hypothesis, which refers to comparing the predicted results to known facts, is a less popular method in PHM for evaluating the model’s performance. An algebraic equation representing prior information is typically used by the learning algorithm to attach a loss term to the model’s loss function, placing restrictions on the model. Inspired by Rueden’s paper, von Hahn et al. [29] represented the knowledge of bearings in reliability engineering by a Weibull cumulative distribution function and integrated the knowledge into ML models by training models based on Weibull’s loss function for the first time. Hahn used the simplest feature processing and model architecture to experiment on the IMS [30] and PRONOSTIA [31] datasets, and the Weibull-combined loss function performed well on most of the datasets, especially the IMS dataset, which significantly increased the accuracy of the RUL prediction and confirmed the viability and effectiveness of the Weibull-based loss function.

On one hand, ML-based aero-engine RUL prediction is being studied by combining different neural networks to construct hybrid models or by using the most recent algorithmic models to improve prediction accuracy, such as Li et al. [32], Liu et al. [33], Zhao et al. [34], and others. On the other hand, because of the multi-serial and time-series nature of the aero-engine dataset, many studies, such as Jiang et al. [35], Wang et al. [36], and Zhang et al. [18], have attempted to make the model more focused on important information and achieve long time series prediction by introducing SA and transformer structures. As a result, a lot of effort is spent on building complex ML models, and the domain knowledge of the devices is neglected and not fully utilized.

Based on the above issues and inspired by the work of Rueden and von Hahn, this paper proposes a new multiform IML prediction model and applies it to the remaining life prediction of aero-engines. The main contributions are as follows:

(1) According to the IML taxonomy, this paper introduces the approach and process of integrating knowledge into machine learning models for the RUL prediction of the aero-engine from three perspectives: knowledge source, knowledge representation, and knowledge integration. Scientific knowledge in reliability engineering of aero-engines is expressed formally as two forms, Piecewise RUL and Weibull CDF, which are integrated into the machine learning model process via training data and learning algorithms, respectively.

(2) Three different loss functions combining Weibull CDF were constructed, and an IML model based on two forms of knowledge was built. The parameters of the Weibull CDF were determined by analyzing the existing knowledge of aero-engine failure probabilities and the available failure time data. Based on conventional loss functions, three new loss functions incorporating Weibull were constructed. The models were trained using Weibull-combined loss functions to output well-trained IML models for RUL prediction.

(3) The domain knowledge was successfully incorporated into the engine RUL prediction model, and the enhancement of the model prediction performance by different forms of knowledge was explored.

The structure of this paper is as follows: The Section 2 introduces the construction process of a multiform IML model and demonstrates the model developed in this paper; the Section 3 describes the data used in the experiments and the settings of the model parameters; the Section 4 confirms the efficacy and superiority of the method by examining the experimental findings; and the Section 5 concludes the paper.

## 2. Methodology

### 2.1. Multiform Informed Machine Learning

This section explains how to build a multiform IML model by combining reliability engineering expertise with ML models. The area of reliability engineering has a wealth of knowledge regarding the deterioration and failure laws of aero-engine equipment that may be integrated into the machine learning pipeline after formal description.

#### 2.1.1. Knowledge Source

The reliability engineering discipline, or the scientific information in the taxonomy, is where the knowledge regarding aero-engines in this study comes from. In the subject of PHM, reliability engineering is frequently utilized as a procedure to avoid, assess, and manage failures [37].

Knowledge 1: Equipment’s early degradation can be negligible until a certain time. If a turbofan engine’s whole life cycle is 192 cycles, the design of its RUL label for aero-engines is 192, 191, 190, …, 1. However, in actual use, a threshold value is frequently employed to ensure that the engine is initially in good functioning condition with no degradation [15,17,38].

**Figure 2:**
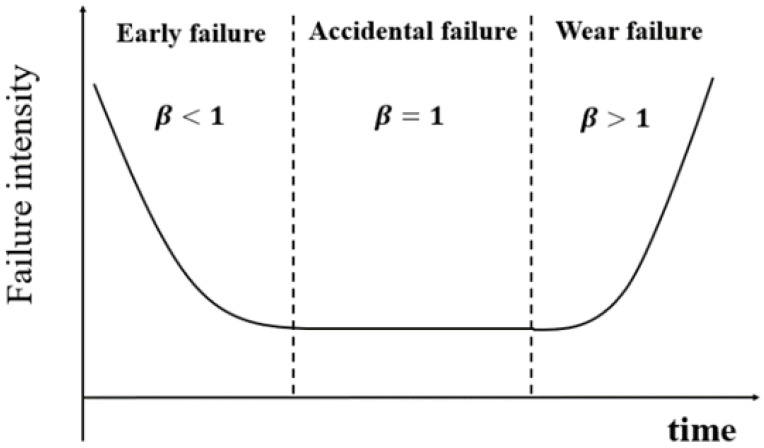
The “bathtub” curve.

Knowledge 2: The failure rate of aero-engine equipment varies over time and follows the law of the bathtub curve [39], which may be represented by the Weibull function. Identifying the causes of equipment failure and describing the equipment failure law is an important task in reliability engineering. For example, the failure rate of most equipment appears as a function of time, with the bathtub curve being the most typical and often utilized. Figure 2 shows a typical bathtub curve diagram. In reliability engineering, equipment failure data are frequently gathered to study the equipment failure pattern. This method was also employed in the current investigation, which will be discussed in Section 2.1.2.

#### 2.1.2. Knowledge Representation

Representation 1: Piecewise RUL

**Figure 3:**
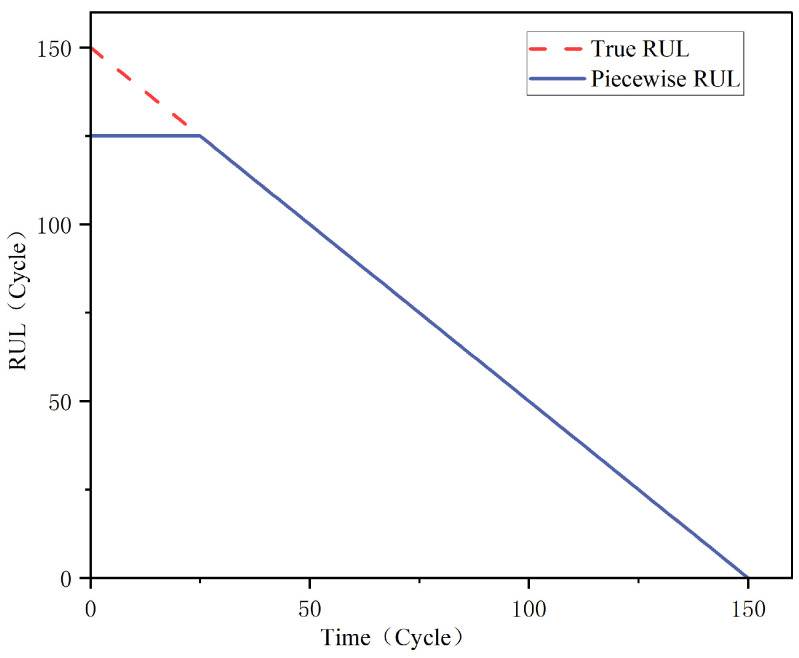
Piecewise RUL Function.

We do not know the RUL of the working healthy equipment or when it starts to deteriorate; therefore, the label setting for the training dataset must also integrate external knowledge. The Piecewise RUL is employed in this paper to set the training set labels because the monitoring data of aero-engines comprise a long time series, and the early performance of aero-engines is relatively stable. As demonstrated in Figure 3, the degradation threshold for an aero-engine is initially set at a fixed maximum RUL ($RUL_{max}$), after which the engine begins to decline linearly. It has been demonstrated that Piecewise RUL is efficient for the C-MAPSS dataset and significantly enhances model performance [14].

Representation 2: Weibull cumulative distribution function

**Figure 4:**
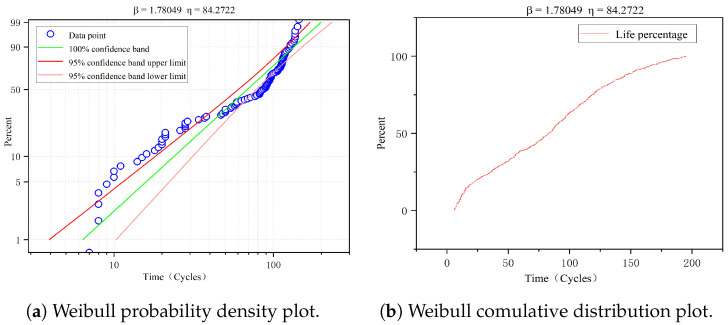
Results of the Weibull fitting for failure time data on FD001.

There is a wealth of literature currently available that examines the use of the Weibull function in many aspects of aero-engines such as design, construction, and PHM. Zaretsky et al. [39] proposed a design method for rotating aircraft engine structures based on a Weibull failure probability analysis. Pascovici et al. [40] performed a lifetime analysis of engine components using the Weibull function to estimate the maintenance cost, direct operating cost, and net present value cost of a future-type turbofan engine. Yuan et al. [41] used a hybrid three-parameter Weibull model to explore the diversity of aero-engine failure modes. Nnaji et al. [42] conducted an extensive Weibull-based data analysis to explore the relationship between engine failure and runtime. Consequently, using the Weibull function to express the external knowledge regarding aero-engine failure in this research is both rational and scientific. According to the practice of von Hahn et al. [29], Weibull’s cumulative distribution function is used in the specific implementation, in the form of Equation (1):

(1) $$F\left(t\right)=1-e^{-{\left(t/\eta \right)}^{\beta }},$$

where *t* is the time, β is the shape parameter, and η is the characteristic lifetime. The specific values of β and η need to be determined by combining the equipment failure time data and the existing knowledge of the device in reliability engineering. In this paper, the failure time data of each sub-dataset are aggregated and fitted separately using the Weibull distribution to obtain the preliminary estimates of β and η, and Figure 4 shows the data analysis process using the sub-dataset FD001 as an example.

The failure time data from the C-MAPSS dataset are fitted with the Weibull distribution, and the results of parameter estimation on four sub-datasets are shown in Table 1. As seen in the table, the ranges of parameters β and η are $\left(1.0,2.0\right)$ and $\left(70,100\right)$. Based on the knowledge about aero-engine failures [39,41,42], particularly the experience of von Hahn et al. [29], the model parameter values β and η are taken as 2.0 and 90, respectively, in this paper.

#### 2.1.3. Knowledge Integration

**Figure 5:**
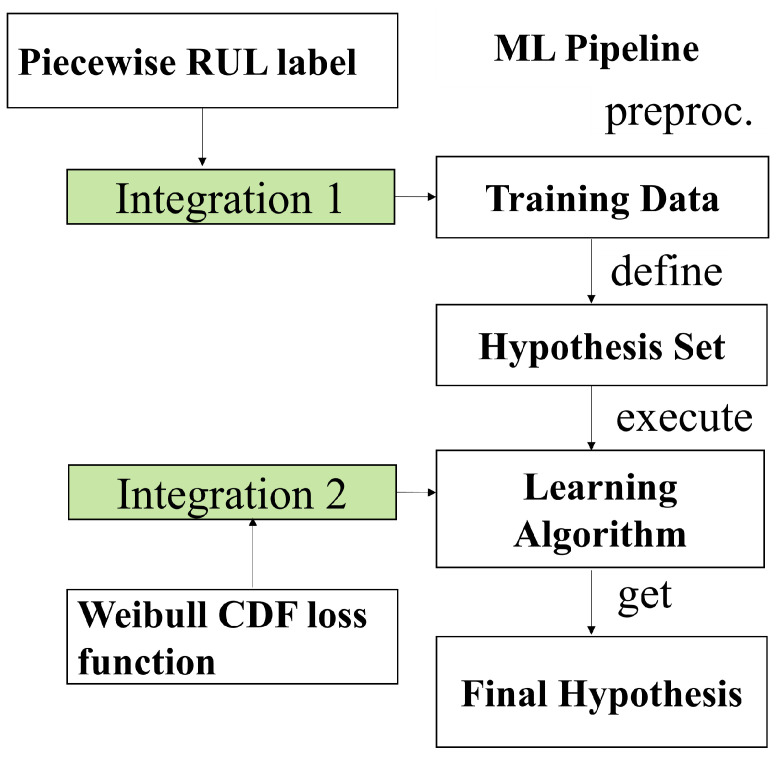
The path of multiform knowledge integrated to the ML pipline.

The final phase in IML is knowledge integration. In this paper, knowledge integration is achieved through training data and learning algorithms, as shown in Figure 5. Knowledge representation 1 is integrated into the training data in the form of labels, and knowledge representation 2 is integrated into the learning algorithm in the form of a Weibull-combined loss function.

Integration 1: Label setting based on Piecewise RUL

When utilizing Piecewise RUL to label the data, the sample’s maximum number of cycles is subtracted from the number of cycles it is presently running to determine its true remaining life ($RUL_{\mathrm{real}\;}$). After that, the Piecewise segmented linear deterioration model is utilized to set the labels ($RUL_{\mathrm{label}\;}$), with the detailed setting approach based on Equation (2).

(2) $$RUL_{\mathrm{label}\;}=\left\{\begin{array}{cc} RUL_{\max}, & RUL_{\mathrm{real}\;}>RUL_{\max\;}\\ RUL_{\mathrm{real}\;}, & RUL_{\mathrm{real}\;}\leq RUL_{\max\;} \end{array}\right..$$

Integration 2: Weibull-combined loss functions

Learning algorithms usually involve modifying the loss function based on external knowledge. By using a Weibull-combined loss function, knowledge is integrated into the loss function for model training, which acts as a constraint on the model. The Weibull-combined loss function is a combination of domain knowledge-based and traditional label-based loss functions, combined in the manner of Equation (3):

(3) $$f^{*}=\arg\min_{f}\left(\overset{\mathrm{Label}\text{-}\mathrm{based}\;}{\overset{︷}{\lambda_{l}\sum_{l}L\left(f\left(x_{l}\right),y_{l}\right)}}+\overset{\mathrm{Regul}.\;}{\overset{︷}{\lambda_{r}R\left(f\right)}}+\underset{\mathrm{Knowledge}\text{-}\mathrm{based}\;}{\underset{︸}{\lambda_{k}L_{k}\left(f\left(x_{i}\right),x_{i}\right)}}\right).$$

The simplified form is Equation (4):

(4) $$L=L_{\mathrm{label}\;}+\lambda L_{\mathrm{domain}\;}$$

where $L_{\mathrm{label}\;}$ is the label-based loss function, such as the commonly used mean square error (MSE) function, root mean square error (RMSE) function, root mean square log error (RMSLE) function, etc. $L_{\mathrm{domain}\;}$ is the domain knowledge-based loss function, such as the Weibull loss function based on the aero-engine reliability knowledge in this paper, and λ is the hyperparameter used to adjust the weight of domain knowledge.

Combining the Weibull CDF $F\left(t\right)$ with a traditional label-based loss function such as the mean square error loss function constitutes a hybrid loss function incorporating Weibull [29], which takes the form of Equation (5):

(5) $$L\left(t,\hat{t}\right)=\underset{\mathrm{MSE}\;\mathrm{loss}\;}{\underset{︸}{\frac{1}{n}\sum_{i=1}^{n}{\left(t_{i}-{\hat{t}}_{i}\right)}^{2}}}+\underset{\mathrm{Weibull}\;\mathrm{loss}\;}{\underset{︸}{\lambda \frac{1}{n}\sum_{i=1}^{n}{\left(F\left(T_{i}-t_{i}\right)-F\left(T_{i}-{\hat{t}}_{i}\right)\right)}^{2}}}$$

where *n* is the number of units in the dataset, $t_{i}$ is the actual remaining life, ${\hat{t}}_{i}$ is the predicted remaining life, $T_{i}$ is the lifetime of the unit, $F\left(T_{i}-t_{i}\right)$ is the failure probability of the unit at the time $T_{i}-t_{i}$, calculated from the Equation (1), and $F\left(T_{i}-{\hat{t}}_{i}\right)$ is the estimated failure probability.

There are three types of Weibull loss functions corresponding to MSE, RMSE, and RMSLE, and the expressions of the three Weibull loss functions are listed in Table 2.

In general, the Weibull loss function is used in combination with other common loss functions. The loss functions in Table 3 are examined experimentally in this study, and their performance in multiple datasets is discussed.

### 2.2. The Proposed Model

**Figure 6:**
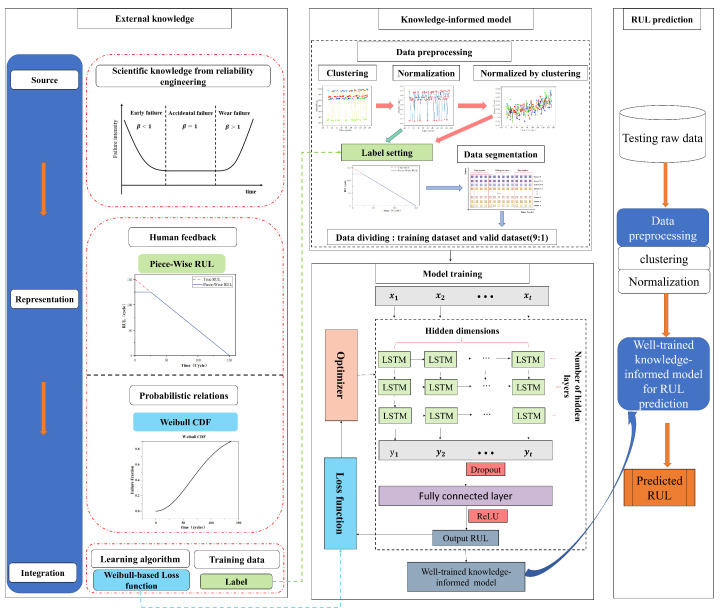
Framework for IML with multiform knowledge.

This research will not use laborious feature extraction engineering or intricately organized ML models because its major goal is to explore the use of IML for engine RUL prediction. The framework of the multiform IML model developed in this paper is illustrated in Figure 6. It is composed of three main components: external knowledge, IML model, and RUL prediction, which are represented by the left part, middle part, and right parts of Figure 6, respectively.

(1) External knowledge: This part, which corresponds to the left side of Figure 6, explains the methods and process of knowledge integrated into machine learning models, which is primarily based on IML taxonomy from knowledge source, knowledge representation, and knowledge integration. The external knowledge in this paper comes from the field of reliability engineering. On the one hand, the device’s early deterioration is minimal, and it begins to degrade at a specific point in time. This formalized degradation function, known as the Piecewise segmented linear degradation function, is recorded as knowledge representation 1. Knowledge representation 1 is used to set the labels of the training and validation sets to achieve knowledge integration. On the other hand, the failure law of most equipment conforms to the bathtub curve in the field of reliability engineering, and the parameter β of the bathtub curve corresponds to the shape parameter β in the Weibull function, so the knowledge above is expressed in the form of the Weibull CDF, which is denoted as knowledge representation 2. Knowledge representation 2 enables knowledge integration by building a loss function combining Weibull CDF. The above two types of knowledge are represented in the left part of Figure 6 with the same color and shape to show the correspondence between the pieces of knowledge.

(2) IML model: This part describes the construction of an informed machine learning model based on the external knowledge in part (1), and the output of a well-trained IML model using the training and validation sets, corresponding to the middle part of Figure 6. Firstly, knowledge representation 2 in part (1) is integrated into the loss function of the machine learning model with a Weibull-combined loss function, completing the construction of the IML model, a relationship indicated by the dashed blue arrows at the bottom of the left part and the middle part in Figure 6. A detailed description of the constructed model is given in Section 3.2 of the article. To create a well-trained IML model with good performance, the model must be trained with data. Before the data are input to the model, they must be preprocessed. Datasets with a single operation condition can be immediately normalized; however, data with multiple operation conditions need to be condition-recognized by K-means clustering and then normalized. The above two forms of processing are denoted by dashed green and red arrows in the data preprocessing section of Figure 6, respectively. The standardized data are labeled according to knowledge representation 1, and then they can be input to the model after the time window processing and dataset division. Eventually, a well-trained multiform IML model is output.

(3) RUL prediction: This section focuses on applying the test dataset to the weill-trained IML model from part (2) so as to predict RUL. The data preprocessing procedure is the same as for the training set.

## 3. Datasets and Experimental Setting

### 3.1. Datasets

The turbofan engine degradation simulation (C-MAPSS) dataset is one of the most popular public datasets provided by NASA for RUL prediction [20]. The C-MAPSS dataset consists of four different sub-datasets, each of which includes data collected from 21 sensors, as shown in Table 4. Each sub-dataset has different operating conditions, with FD002 and FD004 being the most complex. Therefore, the RUL prediction for FD002 and FD004 is more difficult because operating conditions are directly related to the sensor data.

### 3.2. Data Preprocessing

#### 3.2.1. Condition Recognition and Normalization

(1) Condition recognition

The circumstances that engines typically operate under are complicated and varied, and the data produced are both closely related and distinct. However, ML requires independent samples with the same distribution, which cannot be met in the aforementioned situation. When data from different conditions are modeled individually, this may result in insufficient training samples or information loss because of breaking serial correlations. As a result, in this study, data with diverse operating conditions are first clustered using K-means and then normalized based on the clustering result, reducing or even eliminating the influence of varied operating conditions on the model.

**Figure 7:**
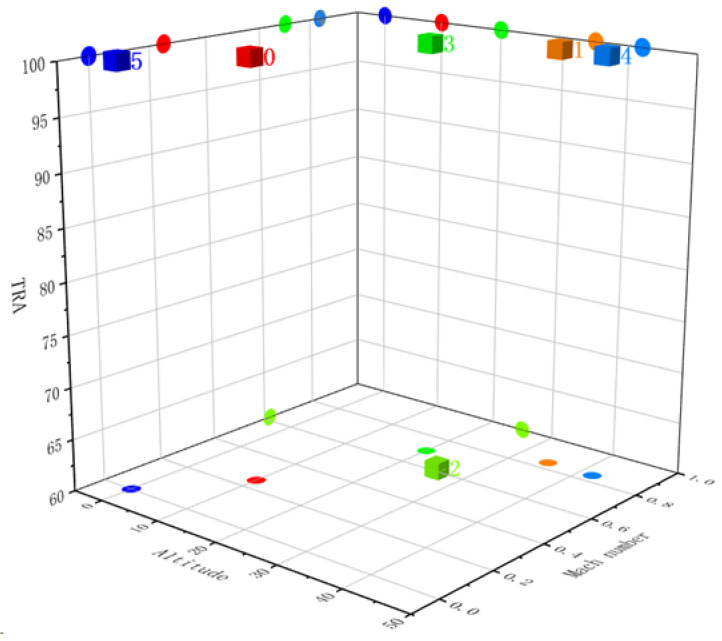
Clustering result of operation condition on FD002.

**Figure 8:**
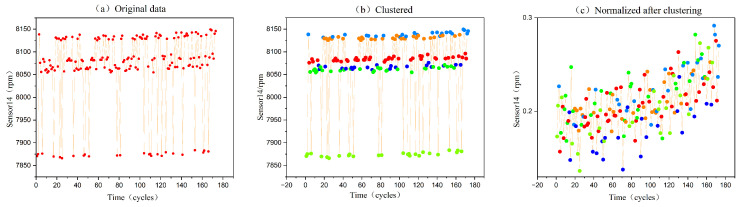
Processing trajectory for the 14th sensor of the 8th engine in the FD002 dataset.

Figure 7 shows the clustering results for the FD002, where different categories are distinguished by different colored cubes, and the operation condition to which the category belongs is labeled with a number. To show the clustering results more clearly, the projections of each category in the XY, XZ, and YZ planes are also drawn in the figure and are shown in Figure 8 as circular icons with the same color as the categories.

(2) Normalization

The result of condition recognition is expressed as ${\left\{X\right\}}^{k}$, which means the $k_{th}$ operation condition; ${\left\{X_{i,t}\right\}}^{k}$ indicates that the data of the $i_{th}$ sensor at moment *t* belong to the $k_{th}$ operation condition; $\min\left({\left\{X_{i,t}\right\}}^{k}\right)$ and $\max\left({\left\{X_{i,t}\right\}}^{k}\right)$ show the maximum and minimum values in a certain operation conditions.

The C-MAPSS data need to be de-unitized because they come from 21 sensors with various scales and units. The maximum–minimum normalization is employed in this research to handle the data. For the multi-source sensor data in the C-MAPSS dataset $X_{i}=\left\{X_{i,1},X_{i,2},\cdots ,X_{i,t}\right\}$, where $X_{i,t}$ represents the data of the $i_{th}$ sensor at the moment *t*, it is normalized using the following equation:

(6) $$\hat{X_{i,t}}=\frac{X_{i,t}-\min\left({\left\{X_{i,t}\right\}}^{k}\right)}{\max\left({\left\{X_{i,t}\right\}}^{k}\right)-\min\left({\left\{X_{i,t}\right\}}^{k}\right)}$$

where $\hat{X_{i,t}}$ is the standardized data in range of [0:1]. Figure 8 depicts the data processing flow and associated outcomes for the 14th sensor of the 8th engine in FD002. Figure 8a displays the original data as a dotted line plot; Figure 8b shows the result of clustering the data according to the operating conditions, which are represented by six coloured dots, each colour corresponding to an operating condition; Figure 8c shows the normalization results separately by the condition recognition, which demonstrates that the original data show a trend of correlation after condition recognition and normalization.

#### 3.2.2. Time Window

**Figure 9:**
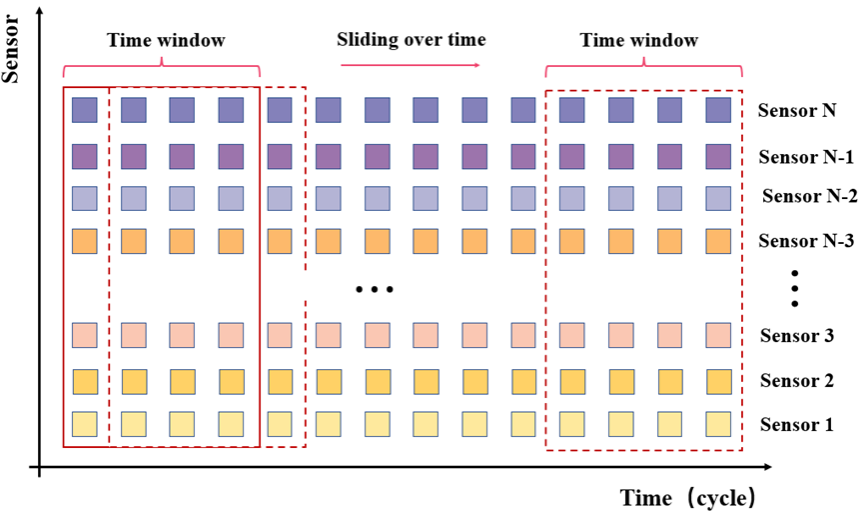
Sliding window processing.

The sliding window technique is typically used to separate the data in multivariate time series, which capture the dependencies between data by using different time steps. A straightforward example of sliding time window processing is shown in Figure 9. A good choice of the window length is required, since an excessively long time window may complicate the model’s structure and limit its application, while an excessively small window will not adequately capture the relationships in the time series.

### 3.3. Experimental Setting

The parameters in this work are divided into two categories: knowledge-based parameters and ML parameters. The knowledge-based parameters are a shape parameter (β), the characteristic lifetime (η), the parameter (λ) to adjust knowledge weight in the Weibull-combined loss function, and ${RUL}_{max}$ in Piecewise RUL. Based on domain knowledge in reliability engineering, parameters of the Weibull-combined loss function have been determined in the previous section. ${RUL}_{max}$ is set based on the experience of Li et al. [21] and Zhang et al. [22], and the effect of different ${RUL}_{max}$ on the model is discussed in Section 4.2. Table 5 shows the settings of the above parameters.

In this paper, experiments were conducted on four sub-datasets of the C-MAPSS dataset, and the grid search method was used to select the model hyperparameters. The LSTM was used as the hidden layer neuron in the model, the activation function was ReLU, and the number of hidden layers was 100. The length of the sliding window was 30. The Adam optimization algorithm was used as the optimizer, and the learning rate was 0.0002. Model batch size and loss rate were 128 and 0.5, respectively. The precise settings of the ML parameters are provided in Table 6.

### 3.4. Evaluation Metrics

The paper employs RMSE and Score metrics, two widely used performance indicators, to evaluate the model’s performance.

(1) RMSE is a common evaluation metric in RUL prediction and is defined in Equation (7):

(7) $$RMSE=\sqrt{\frac{1}{N}\sum_{i=1}^{L}{\left(y_{i}^{\mathrm{pre}}-y_{i}^{\mathrm{true}\;}\right)}^{2}}$$

where N is the number of units and $y_{i}^{\mathrm{pre}}$ and $y_{i}^{\mathrm{true}\;}$ are the predicted and actual RUL of the unit.

**Figure 10:**
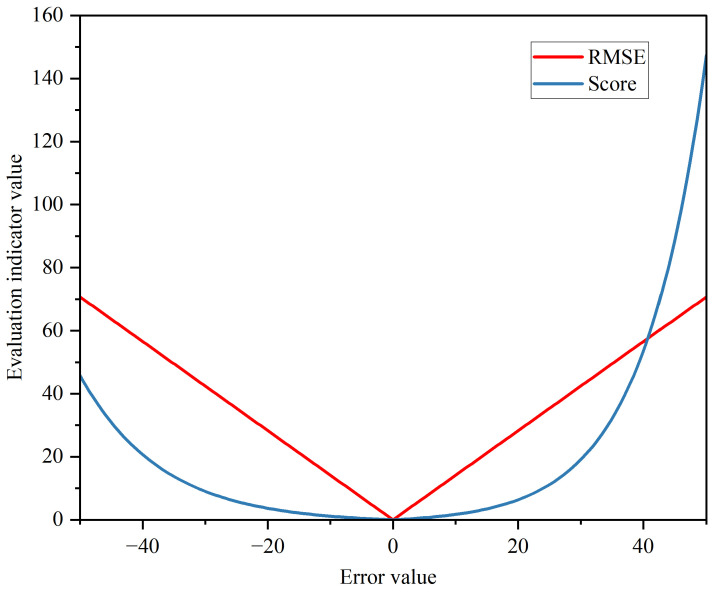
Comparison of RMSE and Score metrics.

(2) In the form presented in Equation (8), the scoring metric that was proposed for the PHM competition has been widely used [2,18,19]. The characteristics and differences of the two evaluation metrics are depicted in Figure 10. The Score metric has a bigger weight of penalty for a prediction exceeding the actual value, which is because lagging predictions can result in serious accidents, whereas RMSE has the same weight of penalty for over-predictions and trailing predictions.

(8) $$\;\mathrm{Score}\;=\left\{\begin{array}{cc} \sum_{i=1}^{N}e^{-\left(\frac{y_{t}^{\mathrm{pre}\;}-y_{i}^{\mathrm{tue}\;}}{13}\right)}-1 & \;\mathrm{for}\;y_{i}^{\mathrm{pre}\;}-y_{i}^{\mathrm{true}\;}<0\\ \sum_{i=1}^{N}e^{\left(\frac{y_{t}^{\mathrm{pre}\;}-y_{i}^{\mathrm{tue}\;}}{10}\right)}-1 & \;\mathrm{for}\;y_{i}^{\mathrm{pre}}-y_{i}^{\mathrm{true}\;}\geq 0 \end{array}\right.$$

## 4. Results Analysis

This section investigates and analyzes the performance of the multiform IML model by using the RMSE and Score metrics introduced in Section 3.3. To ensure the accuracy and reliability of the experimental results, all experiments were repeated 15 times.

### 4.1. Ablation Study

**Figure 11:**
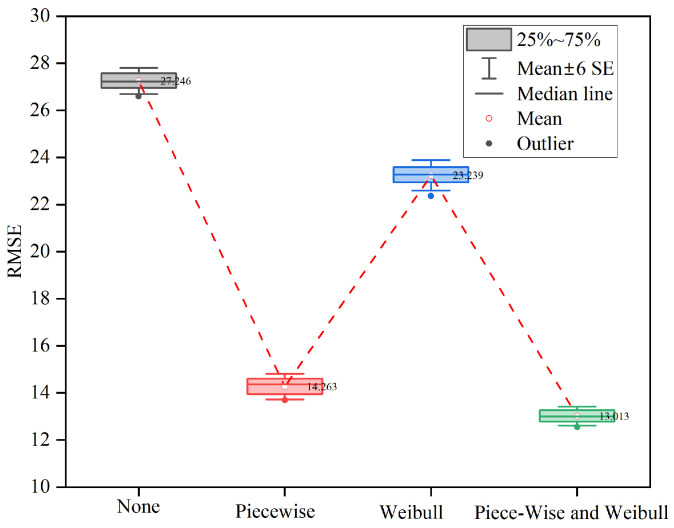
Ablation study on FD002.

Ablation experiments were designed using the FD002 dataset as an example to investigate how the two types of knowledge, Piecewise RUL and Weibull, might improve the model’s prediction accuracy. Four scenarios were considered for the experiment: “Neither Piecewise nor Weibull was considered”, “Piecewise only”, “Weibull only”, and “both Piecewise and Weibull”, corresponding to “None”, “Piecewise”, “Weibull”, and “Piecewise and Weibull” in the horizontal coordinates of Figure 11. Using “None” as the benchmark, the RMSE of “Piecewise” and “Weibull” was reduced by about 13 and 4, respectively, and “Piecewise and Weibull” was reduced the most, by 14.2. This suggests that both types of knowledge increase the model prediction’s accuracy and that the combination of both types of knowledge has the greatest effect on model performance improvement.

Based on the above experimental results, it can be inferred that Piecewise provides semantic information and business meaning to the training data by setting labels, which help the model evaluation and application, and that Weibull provides direction for the model optimization by fitting the failure data distribution, which helps the model fit the data better. Both kinds of knowledge have a positive effect on the model, so the prediction effect is improved significantly after integrating them.

### 4.2. Impact Analysis of the Multiform Knowledge

#### 4.2.1. Impact of Piecewise RUL

**Figure 12:**
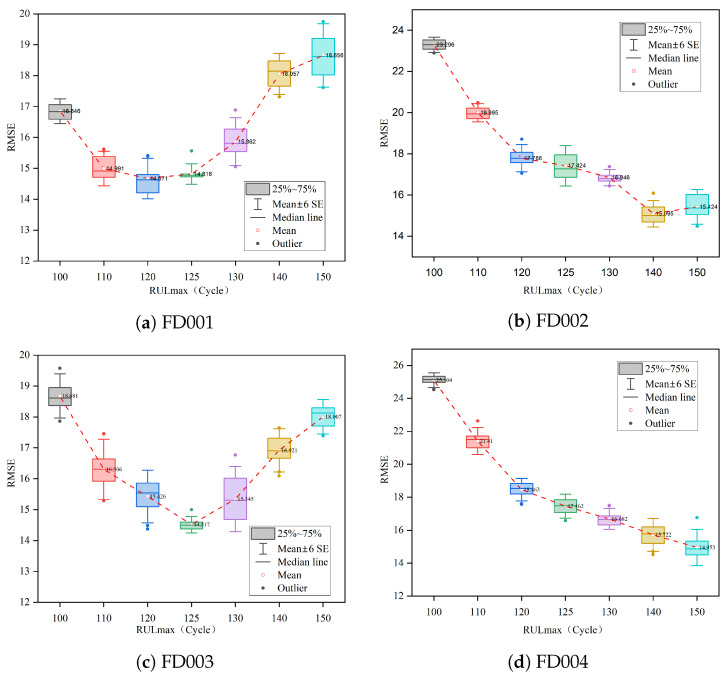
Impact analysis of Piecewise RUL.

This research performed tests by changing the value of $RUL_{max}$ to evaluate the impact of Piecewise RUL on the model. Figure 12 displays a boxplot of the RMSE for the prediction results. For datasets FD001 and FD003, the box height is the lowest, and the mean value is the smallest, with a $RUL_{max}$ around 125, indicating that the model is currently more stable and accurate at predicting RUL. For datasets FD002 and FD004, they have relatively good model stability at a RUL of approximately 130, which is consistent with the results in other studies [15,17,38]. For datasets FD002 and FD004, the model has relatively good stability at a $RUL_{max}$ of 130.

#### 4.2.2. Impact of Weibull

**Figure 13:**
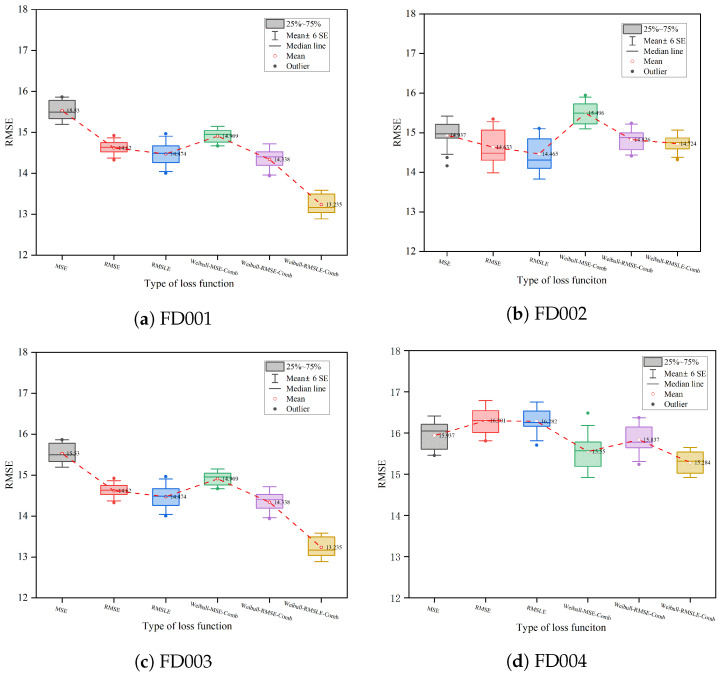
Impact analysis of Weibull.

In order to investigate the impact of Weibull on the model, experiments were carried out with six loss functions on each sub-dataset. The statistical results of the experiments are displayed in Figure 13. The figure shows that, on most of the sub-datasets, the mean of the RMSE and the width of the boxplot of the model incorporating the Weibull loss function are smaller than those of the model based on the conventional loss function, indicating that the model combining Weibull is more stable and its prediction results are more accurate. The Weibull–RMSLE–Comb model has the best prediction accuracy among the datasets FD001, FD003, and FD004 and, even though it is the second-best model in FD002, it has the best model stability because the predicted results have the narrowest range of RMSE distribution. The above analysis demonstrates that the loss function that incorporates Weibull has an effect on the accuracy and stability of model RUL prediction, with the Weibull–RMSLE–Comb model being the most stable and best-performing model.

From the above analysis, we can infer that the Weibull-combined loss function provides direction for the model optimization by fitting the failure data distribution, which helps the model fit the data better. By incorporating the Weibull function into the loss function, we can effectively reduce the prediction error and improve the model robustness.

### 4.3. Analysis of RUL Prediction Results

**Figure 14:**
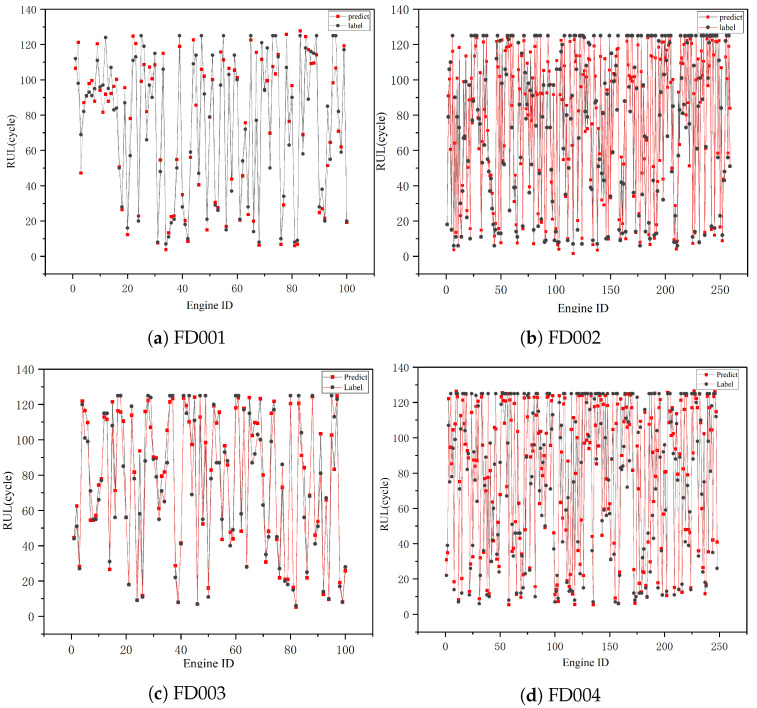
RUL predictions of all engines on four sub-datasets.

Figure 14 displays the prediction values compared to actual values of all test units for the four sub-datasets to highlight the prediction effect of the knowledge-based model. Figure 14 shows that the predicted RUL of the knowledge-based model is similar to the actual RUL and that it outperforms FD001 and FD003 for the FD002 and FD004. However, because of the complexity of the operating conditions, FD002 and FD004 are more challenging to predict in reality, which to some extent suggests that the multiform knowledge-based model has good application prospects and impacts in the case of complex operating conditions.

### 4.4. Comparisons with State-of-the-Art Methods

In comparison to datasets FD001 and FD003, the RUL of datasets FD002 and FD004 is more challenging to estimate due to more complex actual operating conditions. Hence, using the FD002 and FD004 datasets, this research compares the prediction results of the constructed multiform IML model with those of the proposed models from 2017. In Table 7, the RMSE and Score of the various models are displayed along with the associated mean values and a bolding of the top-performing models. Table 7 shows that the multiform IML model developed in this study outperforms the previous models in every metric, with the exception of the RMSE of FD004. The RMSE and Score are reduced on average by 1.29% and 12.59% when compared to the most recent model, MSTformer, proving the multiform IML model’s strong prediction ability for complicated operating conditions. Given the properties of the Score function and the fact that the Score is substantially lower in the model developed in this study, it can be assumed that this is because the model makes more advanced predictions than lagging predictions. It shows that the multiform IML model is more conservative in estimating the RUL as a result of the inclusion of knowledge of reliability engineering, which enhances the predictability and accuracy of the prediction.

## 5. Conclusions and Future Work

This research proposed and applied a novel multiform knowledge-based IML model for the RUL prediction of aero-engines. The dataset labels were set using the aero-engine degradation knowledge represented by Piecewise RUL, and the engine failure probability knowledge was incorporated into the model using a Weibull-combined loss function. Without arduous data pre-processing and sophisticated model design, the degradation features in the data can be captured more precisely with the aid of external knowledge, and the RUL prediction performance of the model under challenging operating conditions can be enhanced. For the FD002 and FD004 datasets, the Piecewise and Weibull-based multiform IML models perform well, with an average improvement in RMSE and Score of 1.29% and 12.59%, respectively, over the best approach currently available, MSTformer. Future research will focus on incorporating reliability engineering domain knowledge into ML models in the form of simulated data, which should address the problem of sparse data making it challenging to support the training of ML models.

In a future study, we will investigate the application of machine learning models to the prediction of the remaining life of equipment using more complicated types of domain knowledge, such as physical models of equipment and degradation mechanisms.

## Figures and Tables

**Table 1:** Estimation results for the Weibull parameter.

Sub-Dataset	Parameter	Estimation	95% Lower Confidence Limit	95% Upper Confidence Limit
FD001	β	84.272	75.123	94.535
	η	1.780	1.503	2.109
FD002	β	88.775	81.134	97.136
	η	1.418	1.281	1.570
FD003	β	84.193	75.157	94.314
	η	1.805	1.529	2.130
FD004	β	95.389	87.494	103.997
	η	1.509	1.359	1.675

**Table 2:** Weibull loss function.

Loss Function	Equation
Weibull Only MSE loss ($L_{\mathrm{Weibull}\text{-}\mathrm{MSE}}$)	$\lambda \frac{1}{n}\sum_{i=1}^{n}{\left(F\left(T_{i}-t_{i}\right)-F\left(T_{i}-{\hat{t}}_{i}\right)\right)}^{2}$
Weibull Only RMSE loss ($L_{\mathrm{Weibull}\text{-}\mathrm{RMSE}}$)	$\lambda \sqrt{\frac{1}{n}\sum_{i=1}^{n}{\left(F\left(T_{i}-t_{i}\right)-F\left(T_{i}-{\hat{t}}_{i}\right)\right)}^{2}}$
Weibull Only RMSLE loss ($L_{\mathrm{Weibull}\text{-}\mathrm{RMSLE}}$)	$\lambda \sqrt{\frac{1}{n}\sum_{i=1}^{n}{\left(\log\left(F\left(T_{i}-t_{i}\right)+1\right)-\log\left(F\left(T_{i}-{\hat{t}}_{i}\right)+1\right)\right)}^{2}}$

**Table 3:** The loss functions tested in the experiment.

Loss Function	Equation
MSE loss ($L_{\mathrm{MSE}}$)	$\frac{1}{n}\sum_{i=1}^{n}{\left(t_{i}-\hat{t_{i}}\right)}^{2}$
RMSE loss ($L_{\mathrm{RMSE}}$)	$\sqrt{\frac{1}{n}\sum_{i=1}^{n}{\left(t_{i}-{\hat{t}}_{i}\right)}^{2}}$
RMSLE loss ($L_{\mathrm{RMSLE}}$)	$\sqrt{\frac{1}{n}\sum_{i=1}^{n}{\left(\log\left(t_{i}+1\right)-\log\left({\hat{t}}_{i}+1\right)\right)}^{2}}$
Weibull–MSE Combined ($L_{\mathrm{Weibull}\text{-}\mathrm{MSE}\text{-}\mathrm{Comb}}$)	$L_{\mathrm{MSE}\;}+L_{\mathrm{Weibull}\text{-}\mathrm{MSE}\;}$
Weibull–RMSE Combined ($L_{\mathrm{Weibull}\text{-}\mathrm{RMSE}\text{-}\mathrm{Comb}}$)	$L_{\mathrm{RMSE}\;}+L_{\mathrm{Weibull}\text{-}\mathrm{RMSE}\;}$
Weibull–RMSLE Combined ($L_{\mathrm{Weibull}\text{-}\mathrm{RMSLE}\text{-}\mathrm{Comb}}$)	$L_{\mathrm{RMSLE}}+L_{\mathrm{Weibull}\text{-}\mathrm{RMSLE}}$

**Table 4:** Details of C-MAPSS dataset.

Dataset	C-MAPSS			
	FD001	FD002	FD003	FD004
Training engines	100	260	100	249
Testing engines	100	259	100	248
Operating conditions	1	6	1	6
Fault modes	1	1	2	2

**Table 5:** Knowledge-based parameter setting.

Parameter	Value
β	2
η	90
λ	Floating point between 0 and 2
${RUL}_{max}$	100,110,120,125,130,140,150

**Table 6:** ML parameter settings.

Item	Settings
Length of time window	30
Number of features	24
Cell	LSTM
Number of hidden layers	3
Number of neurons in hidden layer	100
Dropout rate	0.5
Max RUL of Piecewise RUL	100,110,120,125,130,140,150
Max epochs	100
Learning rate	0.0002

**Table 7:** Comparison with other methods.

Methods	Year	RMSE			Score		
		FD002	FD004	Average	FD002	FD004	Average
LSTM [14]	2017	24.49	28.17	26.33	4450	5550	5000
Bi-LSTM [15]	2018	23.18	24.86	24.02	4130	5430	4780
GNMR [19]	2020	20.85	21.34	21.10	3196	2795	2996
DA-TCN [17]	2020	16.95	18.23	17.59	1842	2317	2080
AGCNN [33]	2020	19.43	21.5	20.47	1492	3392	2442
DAST [18]	2022	15.25	18.36	16.81	924.96	1490.72	1208
BiGRU-TSAM [43]	2022	18.94	20.47	19.71	2264	3610	2937
MSTformer [38]	2023	14.48	**15.03**	14.76	1099	1012	1056
**This paper**		**14.03**	15.10	**14.57**	**876**	**970**	**923**

## Data Availability

Not applicable.
